# A Derived QSAR Model for Predicting Some Compounds as Potent Antagonist against* Mycobacterium tuberculosis*: A Theoretical Approach

**DOI:** 10.1155/2019/5173786

**Published:** 2019-05-02

**Authors:** Shola Elijah Adeniji, Sani Uba, Adamu Uzairu, David Ebuka Arthur

**Affiliations:** Department of Chemistry, Ahmadu Bello University, Zaria, Nigeria

## Abstract

Development of more potent antituberculosis agents is as a result of emergence of multidrug resistant strains of* M. tuberculosis*. Novel compounds are usually synthesized by trial approach with a lot of errors, which is time consuming and expensive. QSAR is a theoretical approach, which has the potential to reduce the aforementioned problem in discovering new potent drugs against* M. tuberculosis*. This approach was employed to develop multivariate QSAR model to correlate the chemical structures of the 2,4-disubstituted quinoline analogues with their observed activities using a theoretical approach. In order to build the robust QSAR model, Genetic Function Approximation (GFA) was employed as a tool for selecting the best descriptors that could efficiently predict the activities of the inhibitory agents. The developed model was influenced by molecular descriptors: AATS5e, VR1_Dzs, SpMin7_Bhe, TDB9e, and RDF110s. The internal validation test for the derived model was found to have correlation coefficient (R^2^) of 0.9265, adjusted correlation coefficient (R^2^ adj) value of 0.9045, and leave-one-out cross-validation coefficient (Q_cv^∧^2) value of 0.8512, while the external validation test was found to have (R^2^ test) of 0.8034 and Y-randomization coefficient (cR_p^∧^2) of 0.6633. The proposed QSAR model provides a valuable approach for modification of the lead compound and design and synthesis of more potent antitubercular agents.

## 1. Introduction

Tuberculosis (TB) is the most deadly bacterial disease caused by specie of bacteria known as* Mycobacterium tuberculosis*. In 2013, World Health Organization (WHO) estimated death of 1.5 million people, 9.0 million people living with tuberculosis, and 360,000 people who were HIV positive [1]. At present, pyrazinamide (PZA), para-amino salicylic acid (PAS), isoniazid (INH), and rifampicin (RMP) are the current drugs administered to patients suffering from tuberculosis.

The resistance of the* M. tuberculosis* to the current drugs led to development of new approach that is fast and precise and could be able to predict the biological activity for the new compounds against* M. tuberculosis*.

Meanwhile, a theoretical approach, quantitative structure activity relationships (QSARs), is one of the most widely used computational method which helps in designing drugs and predicting drugs activities [2]. QSAR model is a mathematical linear equation which relates the molecular structures of the compounds to their biological activities. In this research, a data set of 2,4-diquinoline derivatives which had been synthesized and evaluated as anti-*Mycobacterium tuberculosis* [3] has been selected for QSAR study. Few researchers [4–7] have established relationship between some antitubercular inhibitors like quinolone, chalcone, pyrrole, and 7-methyljuglone using QSAR approach. However, QSAR study has not been established to relate the structures and activities of 2,4-disubstituted quinoline derivatives as potent antitubercular agents. Therefore, this study aimed to establish a valid QSAR model that could correlate the structures of 2,4-diquinoline derivatives and predict their respective activities against* Mycobacterium tuberculosis*.

## 2. Material and Method

### 2.1. Data Set and Data Collection

The derivatives of 2,4-disubstituted quinoline as potent anti-*Mycobacterium tuberculosis* that were used in this research were selected from the literature [3]. The chemical structures alongside with their biological activities of these compounds were presented in Table 1, while the equation below was used to convert the percentage activities to logarithm unit.

(1) pBA=log⁡Molecular  weightg/molDoseg/mol·percentage%100−percentage%

see [5]

### 2.2. Structure Optimization

In order for the molecules to attain a stable conformer at a minimal energy, all the molecules were geometrically optimized with the aid of Spartan 14 V1.1.4 by employing Molecular Mechanics Force Field (MMFF) count to remove strain energy and later subjected to Density Functional Theory (DFT) by utilizing the (B3LYP) basic set [5].

### 2.3. Molecular Descriptor Calculation

Descriptor is a mathematical logic that describes the properties of a molecule based on the correlation between the structure of the compound and its biological activity. Descriptors calculation for all the inhibitory compounds were achieved using PaDEL-Descriptor software V2.20.

### 2.4. Normalization of Data and Pretreatment

The values for the calculated descriptors were normalized using (2) so that each variable will have the same prospect at the inception so as to sway the model [8]:

(2) $$\begin{array}{c} Y=\frac{Y_{1}-Y_{min}}{Y_{max}-Y_{min}} \end{array}$$

where Y_1_ is the descriptor value for each molecule and Y_min_ and Y_max_ are the minimum and maximum value for each descriptors column of Y. After successful normalization of the data, the data were further subjected to pretreatment in order to remove noise and redundant data.

### 2.5. Data Division into Training and Test Set

Kennard and Stone's algorithm approach was employed in this study to divide the data set into two compounds, a training set and a test, in proportion of 70 to 30%. The training set was used to develop the QSAR model while the test was used to confirm the developed model [9].

### 2.6. Development of the Model

Multilinear regression (MLR) approach is a strategy used to develop the QSAR. MLR approach displays a direct relationship between the dependent variable Y (activity) and independent variable X (descriptors). In MLR analysis, the mean of the dependent variable Y relies on X. MLR equation below is used to incorporate more than one independent variable (descriptors) with a single response variable (activity):

(3) $$\begin{array}{c} Y=k_{1}x_{1}+k_{2}x_{2}+k_{3}x_{3}+C \end{array}$$

where Y represents the dependent variable, represent the independent variables, k's are regression coefficients for each *x*, and C is a regression intercept [9].

### 2.7. Generation of QSAR Model and Validation

The combinations of the optimum descriptors for the training set were obtained from the descriptor pool using the Genetic Function Approximation technique. Their anti-lung cancer activities were placed as the last column in their respective spread sheets in Microsoft Excel 2010 which were later imported into the Material Studio software version 8.0 to generate the QSAR model by employing multilinear regression (MLR) approach and to evaluate the internal validation parameters [9].

### 2.8. Determination of Outlier and Influential Molecule (Applicability Domain)

The applicability domain approach was employed for the determination of outlier and influential molecule. Any compound outside the applicability domain space of ±3 is said to be an outlier. To define and describe the applicability domain of the built QSAR models, the leverage *hi* approach was employed and defined as follows [10].

(4) $$\begin{array}{c} hi=X_{i}{\left(\;X^{T}X\;\right)}^{-1}X_{i}^{T} \end{array}$$

*Xi* is training set matrix of *i*. *X* is the n × k descriptor matrix of the training set compound, and *X*^*T*^ is the transpose of the training set (*X*). *X*_*i*_^*T*^ is the transpose matrix *X*_*i*_ used to build the mode. The warning leverage h^*∗*^ is the limit values to check for influential molecule. The warning leverage h^*∗*^ is defined as

(5) $$\begin{array}{c} h^{*}=3\frac{\left(j+1\right)}{m} \end{array}$$

where *j* is the number of descriptors in the built model and *m* is the number of compounds that made up the training set.

### 2.9. Assessment of Y-Randomization

Y-Randomization test is a confirmatory test to show that the developed QSAR model is reliable, strong, and robust and not gotten by chance. This test was performed on the training set data as described by [11]. Multilinear regression (MLR) models were generated by randomly shuffling the dependent variable (activity data) while keeping the independent variables (descriptors) unaltered. It is expected that the developed QSAR model should have significantly low *R*^2^ and Q^2^ values for numbers of trials in order to ascertain that the developed QSAR model is robust. Y-randomization coefficient (c*R*_*p*_^2^) is another important parameter which should be more than 0.5 for passing this test.

(6) $$\begin{array}{c} cR_{p}^{2}=R\times {\left[\;R^{2}-{\left(\;R_{r}\;\right)}^{2}\;\right]}^{2} \end{array}$$

Here c*R*_*p*_^2^ is Y-randomization coefficient, R is correlation coefficient for *Y*-Randomization, and Rr is average ‘R' of random models.

### 2.10. External Validation of the Model

The external validation test for the developed QSAR model was further subjected to Golbraikh and Tropsha criteria listed below:

- |r0^∧^2 − r′0^∧^2| (threshold value < 0.3)
- r^2^ − r_o_^2^/r^2^ (threshold value < 0.1)
- r^2^ − r′_o_^2^/r^2^ (threshold value < 0.1)
- k (threshold value 0.85 ≤ k ≤ 1.15)
- k′ (threshold value 0.85 ≤ k ≤ 1.15) [11, 12]

where r^2^ is the square correlation coefficients of the plot of observed activity against calculated activity values, r_o_^2^ is the square correlation coefficients of the plot of observed activity against calculated activity values at zero intercept, r′_o_^2^ is the square correlation coefficients of the plot of calculated activity against observed activity at zero intercept, k is the slope of the plot of observed activity against calculated activity values at zero intercept, and k′ is the slope of the plot of calculated against observed activity at zero intercept.

### 2.11. Affirmation of the Built Model

The fitting ability, stability, reliability, predictiveness, and robustness of the developed models were evaluated by internal and external validation parameters. The validation parameters were compared with the accepted threshold value for any QSAR model [10–13] shown in Table 6.

## 3. Results and Discussion

A theoretical approach was employed to derive a QSAR model for predicting the activities of 2,4-disubstituted quinoline analogues against* Mycobacterium tuberculosis*. Kennard-Stone algorithm approach employed in this research was able to divide the studied compounds, which comprise 36 compounds, into a training set of 25 compounds and a test set of 11 compounds. The model generated was built on the basis of the training set while validation of the model was accessed by the test set

The best descriptors that could better predict the activities of the inhibitory compounds were selected with the approach of Genetic Function Algorithm (GFA) while multilinear regression (MLR) method was used as modeling technique in generating the QSAR model. GFA-MLR led to selection of five (5) descriptors and four (4) QSAR models.

*Model 1*

(7) pBA=−6.515153698∗AATS5e+0.056593117∗VR1_Dzs−6.230058484∗SpMin7_Bhe+0.016884210TDB7e+0.09232054RDF90i+43.764308643

*Model 2*

(8) pBA=−6.786545678∗AATS7s+0.001940984∗VR1_Dzi+0.057893236∗VR1_Dzs−6.094663684∗SpMin7_Bhe+0.016865898TDB7e+34.25653286

*Model 3*

(9) pBA=−6.580218678∗AATS5e+0.009643767∗VR3_Dzv−5.683009673∗SpMin7_Bhe+0.017884876TDB7e+0.094520749RDF90i+42.534142880

*Model 4*

(10) pBA=−6.77748965∗AATS5e+0.009657829∗VR3_Dzv+0.053288132∗VR1_Dzi−5.596564878∗SpMin7_Bhe−0.09872434RDF90i+78.665320923

The observed activities, calculated activities of the inhibitors, the residual values, and the leverage value for each compound were reported in Table 1. The low residual values between observed activities and calculated activities indicate that the model generated has a high predictive ability. Meanwhile the calculated descriptors for training set and test set in generating model 1 were reported in Table 2 for the purpose of reproducibility.

The names and symbols of each descriptors selected by GFA approach were presented in Table 3. The combination of the selected descriptors (2D and 3D) reported in model 1 indicates that these types of descriptors are able characterize and give better information on the structure of the antitubercular molecules.

Statistics and correlation matrix of the selected descriptors that were reported in model 1 were presented in Table 4. The descriptors were subjected to Variance Inflation Factor (VIF) in order to check for orthogonality. Meanwhile, the VIF values for each descriptor shown in Table 4 were less than 4, which confirms that the descriptors were statistically significant and orthogonal.

The mean effect (ME) and standard regression coefficient (*b*_*j*_^*s*^) values are reported in Table 4 which gives vital information on the effect of each descriptor and the degree of contribution in the developed model. The signs and the magnitude on the mean effects values indicate direction in influencing the activity of a compound and their individual strength. Table 4 represents the P-values of each of the descriptors in the model at 95% confidence level. Therefore the null hypothesis that says there is no association between the descriptors and the activities of the molecules is rejected; thus, the alternative hypothesis that says there is a relationship between the descriptors used in generating the model and the activities of the compounds at p < 0.05 is accepted. The Person correlation coefficients calculated for the descriptors in the model were reported in Table 5. The low correlation coefficients that exist between each descriptor in the model imply that there exists no significant intercorrelation between each descriptor.

External validation and internal validation parameters used to assure that the developed models are stable and robust were reported in Table 6. These parameters were in agreement with the threshold value reported in Table 6 which actually confirmed the robustness and stability of the model. Based on these validation parameters, model one was selected as the optimum model and used to predict the activities of 2,4-disubstituted quinoline derivatives.

The QSAR model generated in this research was compared with the models obtained in the literature [4, 5] as shown below:

(11) pBA=−0.307001458AATS7s+1.528715398nHBint3+3.976720227minHCsatu+0.016199645TDB9e+0.089381479RDF90i−0.107407822RDF110s+4.057082751Ntrain=35,R2=0.92023900,Radj=0.91017400,Qcv2=0.89538600

*and the external validation for the test set was found to be R*^*2*^*pred = 0.8842* [5]

(12) pIC50=−2.040810634∗nCl−19.024890361∗MATS2m+1.855704759∗RDF140s+6.739013671Ntrain=27,R2=0.9480,Radj=0.9350,Qcv2=0.87994

*and R*^*2*^*pred = 0.7690* [4].

From the above models the validation parameters reported in this work and those reported in the literature were all in agreement with the parameters presented in Table 6, which actually confirmed the robustness of the model generated.

Y-Randomization coefficient (c*R*_*p*_^2^) was also conducted and has a significant value of 0.7443, greater than 0.5, which was reported in Table 7 supporting the claim that the model generated is powerful and not inferred by chance.

**Figure 1:**
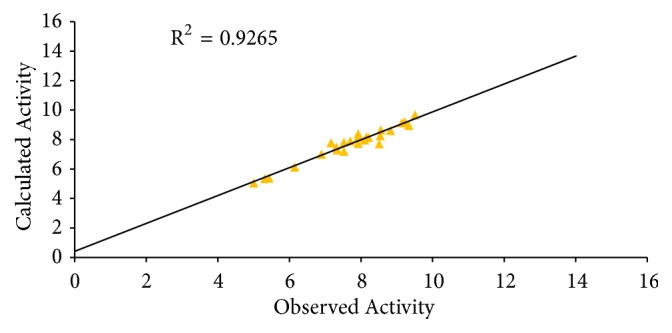
Plot of calculated activity against observed activity of training set.

**Figure 2:**
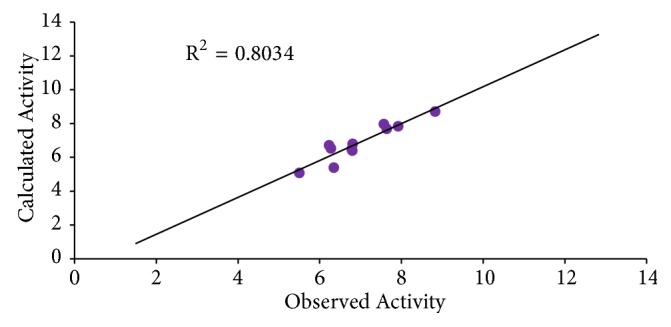
Plot of calculated activity against observed activity of test set.

**Figure 3:**
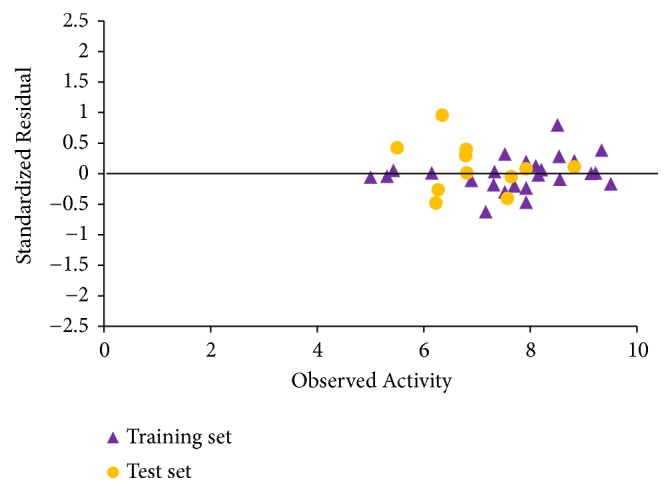
Plot of standardized residual activity versus observed activity.

The graphs of calculated activities plotted against observed activities of the training and test set are presented in Figures 1 and 2. The correlation coefficient (R^2^) value of 0.9265 for the training set and (R^2^) value of 0.8034 for the test set recorded in this work were found to be in line with accepted QSAR threshold values reported in Table 3. This affirms the stability, reliability, and predictive power of the built model. The plot of residual activity against observed activities shown in Figure 3 indicates that there exists no computational inaccuracy in the derived QSAR model as the range of residuals values falls within an accepted limit of ±2 on residual activity axis.

**Figure 4:**
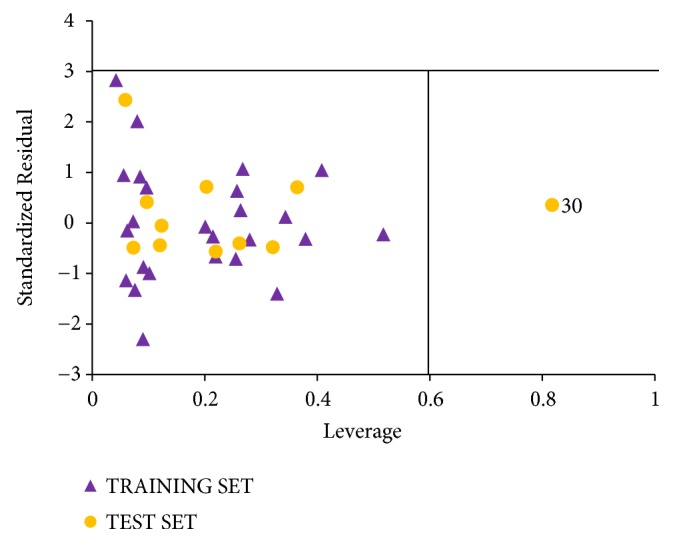
The Williams plot of the standardized residuals versus the leverage value.

The standardized residual activities plotted against the leverage value, known as the Williams plot, are shown in Figure 4. The plotted graph clearly shows that all the compounds fall within limit boundary ±3 of standardized cross-validated residuals. Hence, it can be inferred that no outlier is observed in the data set. However, compound number 30 is found to have a leverage value greater than the calculated warning leverage (h^*∗*^ = 0.60). Therefore the compound is an influential molecule.

### 3.1. D-Optimal Design

D-Optimal design was carried out in order to determine optimal design location and maximize the efficiency of estimating a specified model. This was achieved using Statgraphics 18 software.

From the results presented in Table 8, the R-Squared statistic indicates that the model as fitted explains 80.9278% of the variability in observed activities. The correlation coefficient equals 0.899599, indicating a moderately strong relationship between the variables (descriptors). The standard error of the estimate shows the standard deviation of the residuals to be 0.345508. Thus, value can be used to construct prediction limits for new observations. The mean absolute error (MAE) of 0.25514 is the average value of the residuals. The Durbin-Watson (DW) statistic tests the residuals to determine if there is any significant correlation based on the order in which they occur in the data file. Since the P-value is greater than 0.05, it implies there is no indication of serial autocorrelation in the residuals at the 95.0% confidence level.

**Figure 5:**
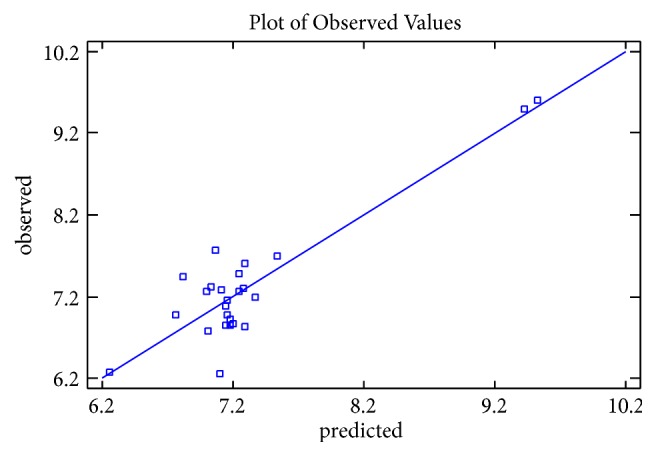
Plot of observed versus predicted values.

**Figure 6:**
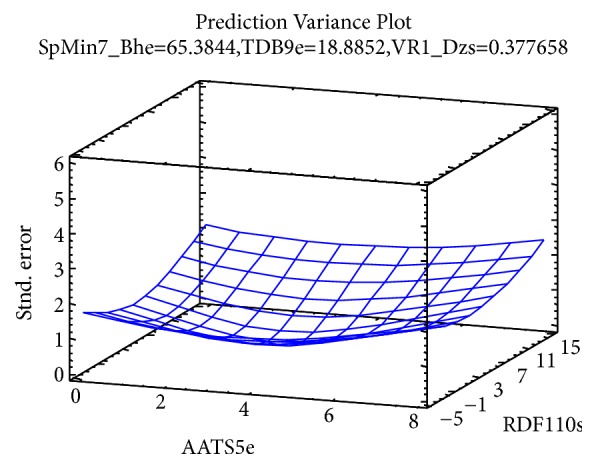
Variance plot shows how the standard error of the predicted response varies across the design region.

**Figure 7:**
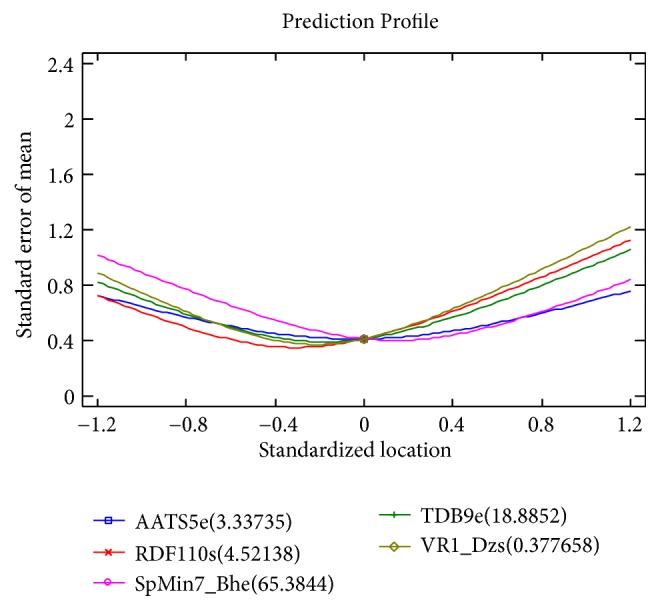
Prediction profile graph displays the standard error of the predicted response.

The* observed versus predicted* plot presented in Figure 5 shows the observed values of Y on the vertical axis and the predicted values of X on the horizontal axis. Based on the fact that the points are randomly scattered around the diagonal line, it indicates that the model fits well. The* Prediction Variance Plot* presented in Figure 6 shows how the standard error of the predicted response varies across the design region. The standard error displayed is the square root of the unscaled prediction variance. A surface plot is created for the first two design factors, AATS5e and RDF110s, with all other factors held constant. In order to have an optimal design, the standard error must be at lowest near the center of the design region. It increases as the location moves away from the center in any direction. The* Prediction Profile* graph presented in Figure 7 displays the standard error of the predicted response as a function of each design factor as the factors are moved from a specified reference point. The location in the design region for each response was AATS5e = 3.34, RDF110s = 4.52, SpMin7_Bhe = 65.38, TDB9e = 18.89, and VR1_Dzs= 0.38, respectively. At these locations, the standard error of prediction equals 0.345508. Therefor the plot shows the location of each factor in standardized units. In standardized units, the specified* low* value equals -0.4, the center is 0, and the specified* high* value equals 0.4. The lines on the plot show how the specified standard error changes as the factors are moved away from the reference location. It can be clearly noticed that the standard errors remain small within the low to high range (-0.4 to 0.4) but start to increase rapidly outside that range.

## 4. Conclusion

A theoretical approach was employed in this study on selected molecular descriptors to derive a model that could be used to correlate the structure of 2,4-disubstituted quinolone derivatives as potent inhibitors against* Mycobacterium tuberculosis* with their respective biological activities. The model derived was subjected to internal and external validation test to confirm that the built QSAR model is significant, robust, and reliable. From the results, it is concluded that 2,4-disubstituted quinolone derivatives can be modeled using molecular descriptors, AATS5e, VR1_Dzs, SpMin7_Bhe, TDB9e, and RDF110s. The built QSAR model will be a vital tool for pharmaceutical as well as medicinal chemists to design and synthesize novel antitubercular drugs with better activities against* M. tuberculosis*.

## Figures and Tables

**Table 1:** Molecular structures of inhibitory compounds and their derivatives as antitubercular agents.

S/N	Molecular structure	Observed Activity (%)	Observed Activity (pA)	Calculated Activity	Residual	Leverage
**1** ^a^	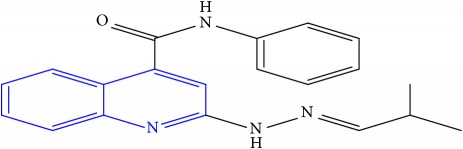 (E)-2-(2-(2-methylpropylidene)hydrazinyl)-N-phenylquinoline-4-carboxamide	11	6.8191	7.22456	-0.40546	0.186966
**2**	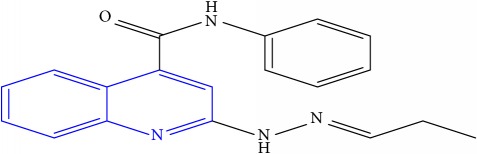 (E)-N-phenyl-2-(2-propylidenehydrazinyl)quinoline-4-carboxamide	12	6.8418	6.713561	0.128239	0.267393
**3**	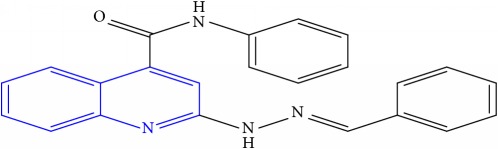 (E)-2-(2-benzylidenehydrazinyl)-N-phenylquinoline-4-carboxamide	11	6.8601	6.664744	0.195356	0.072612
**4**	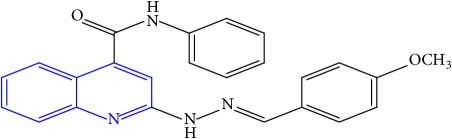 (E)-2-(2-(4-methoxybenzylidene)hydrazinyl)-N-phenylquinoline-4-carboxamide	99	9.4979	9.73193	-0.23403	0.15548
**5**	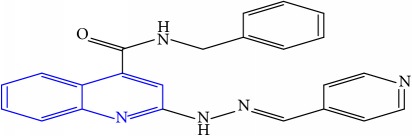 (E)-2-(2-(4-methoxybenzylidene)hydrazinyl)-N-phenylquinoline-4-carboxamide	14	6.9772	6.896778	0.080422	0.328411
**6**	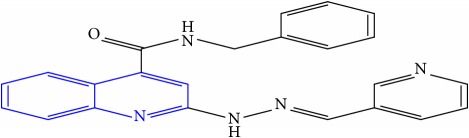 (E)-N-benzyl-2-(2-(pyridin-3-ylmethylene)hydrazinyl)quinoline-4-carboxamide	23	7.2608	6.510442	0.750358	0.055405
**7** ^a^	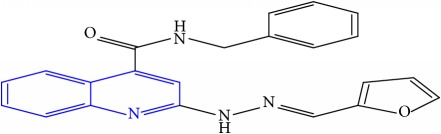 (E)-N-benzyl-2-(2-(furan-2-ylmethylene)hydrazinyl)quinoline-4-carboxamide	20	7.1707	6.972982	0.197718	0.407733
**8** ^a^	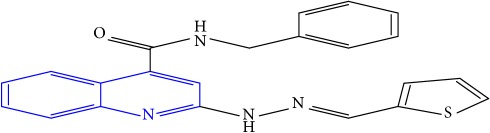 (E)-N-benzyl-2-(2-(thiophen-2-ylmethylene)hydrazinyl)quinoline-4-carboxamide	30	7.4233	7.152527	0.270773	0.378878
**9** ^a^	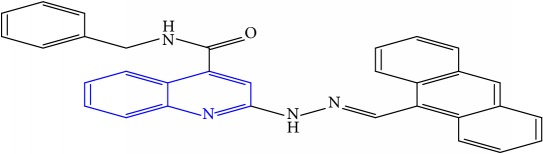 (E)-2-(2-(anthracen-9-ylmethylene)hydrazinyl)-N-benzylquinoline-4-carboxamide	20	7.2838	6.985668	0.298132	0.085176
**10**	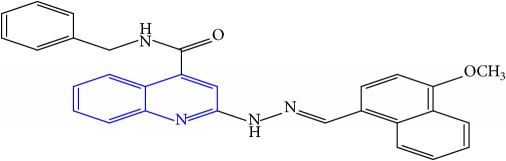 (E)-N-benzyl-2-(2-((4-methoxynaphthalen-1-yl)methylene)hydrazinyl)quinoline-4-carboxamide	16	7.1472	7.67865	-0.53145	0.343511
**11**	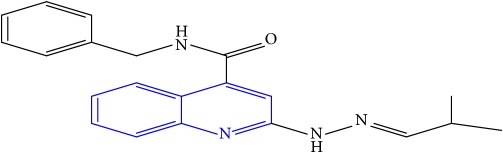 (E)-N-benzyl-2-(2-(2-methylpropylidene)hydrazinyl)quinoline-4-carboxamide	42	7.6035	7.71263	-0.10913	0.084914
**12**	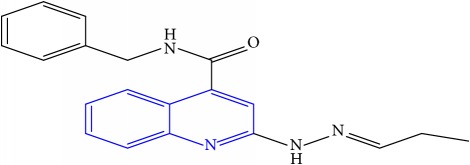 (E)-N-benzyl-2-(2-propylidenehydrazinyl)quinoline-4-carboxamide	27	7.2938	6.495725	0.798075	0.096543
**13**	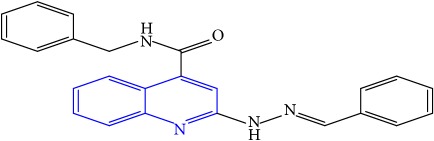 (E)-N-benzyl-2-(2-benzylidenehydrazinyl)quinoline-4-carboxamide	99	9.6090	9.62779	-0.01879	0.089973
**14**	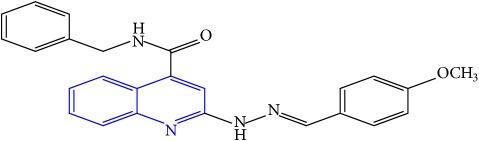 (E)-N-benzyl-2-(2-(4-methoxybenzylidene)hydrazinyl)quinoline-4-carboxamide	21	7.2630	7.88645	-0.62345	0.067538
**15**	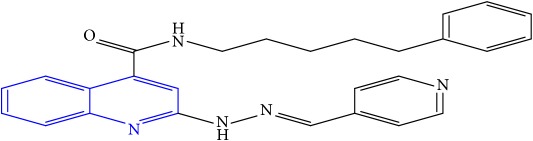 (E)-N-(5-phenylpentyl)-2-(2-(pyridin-4-ylmethylene)hydrazinyl)quinoline-4-carboxamide	30	7.4772	7.411826	0.065374	0.101346
**16** ^a^	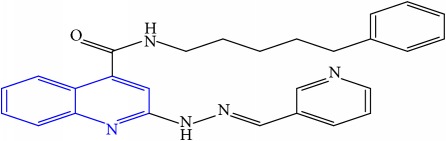 (E)-N-(5-phenylpentyl)-2-(2-(pyridin-3-ylmethylene)hydrazinyl)quinoline-4-carboxamide	10	6.8909	6.781862	0.109038	0.218861
**17**	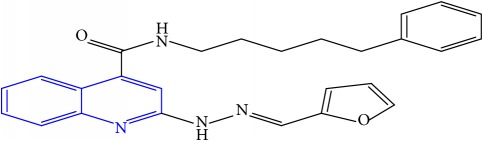 (E)-2-(2-(furan-2-ylmethylene)hydrazinyl)-N-(5-phenylpentyl)quinoline-4-carboxamide	15	7.0807	7.17282	-0.09212	0.090942
**18**	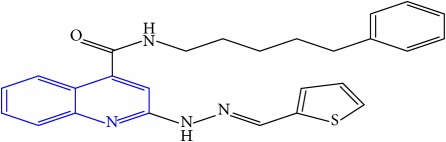 (E)-N-(5-phenylpentyl)-2-(2-(thiophen-2-ylmethylene)hydrazinyl)quinoline-4-carboxamide	21	7.2747	7.224153	0.050547	0.079898
**19** ^a^	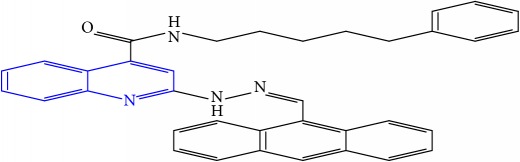 (Z)-2-(2-(anthracen-9-ylmethylene)hydrazinyl)-N-(5-phenylpentyl)quinoline-4-carboxamide	23	7.4091	7.67409	-0.26499	0.075513
**20** ^a^	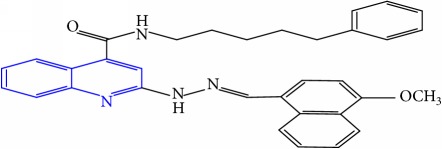 (E)-2-(2-((4-methoxynaphthalen-1-yl)methylene)hydrazinyl)-N-(5-phenylpentyl)quinoline-4-carboxamide	40	7.7412	7.3187	0.4225	0.154686
**21** ^a^	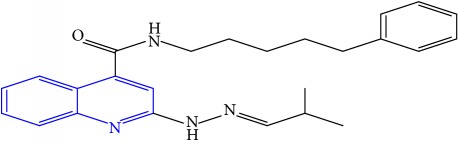 (E)-2-(2-(2-methylpropylidene)hydrazinyl)-N-(5-phenylpentyl)quinoline-4-carboxamide	42	7.6688	7.273758	0.395042	0.0423
**22**	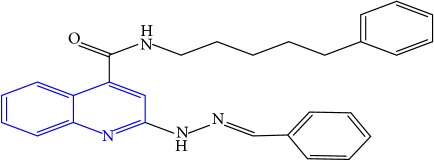 (E)-2-(2-benzylidenehydrazinyl)-N-(5-phenylpentyl)quinoline-4-carboxamide	21	6.2688	6.3256	-0.0568	0.05984
**23**	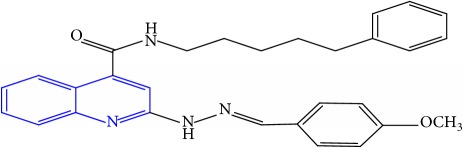 (E)-2-(2-(4-methoxybenzylidene)hydrazinyl)-N-(5-phenylpentyl)quinoline-4-carboxamide	40	7.6970	7.73765	-0.04065	0.357197
**24** ^a^	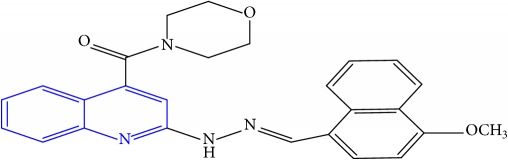 (E)-(2-(2-((4-methoxynaphthalen-1-yl)methylene)hydrazinyl)quinolin-4-yl)(morpholino)methanone	7	6.7741	5.816571	0.957529	0.214607
**25**	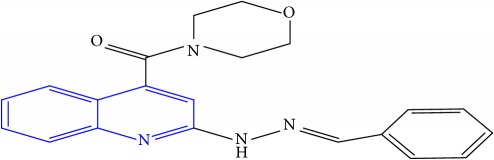 (E)-(2-(2-benzylidenehydrazinyl)quinolin-4-yl)(morpholino)methanone	3	6.2513	6.039603	0.211697	0.200793
**26**	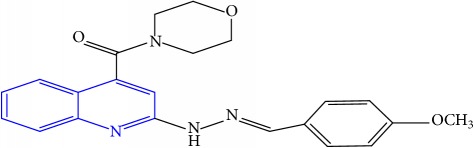 (E)-(2-(2-(4-methoxybenzylidene)hydrazinyl)quinolin-4-yl)(morpholino)methanone	10	6.8414	6.809542	0.031858	0.432707
**27** ^a^	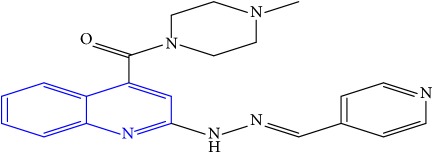 (E)-(4-methylpiperazin-1-yl)(2-(2-(pyridin-4-ylmethylene)hydrazinyl)quinolin-4-yl)methanone	28	7.3673	7.357741	0.009559	0.263698
**28**	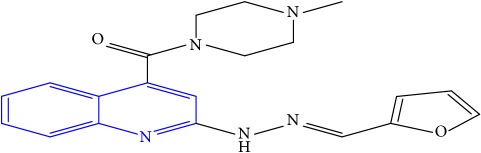 (E)-(2-(2-(furan-2-ylmethylene)hydrazinyl)quinolin-4-yl)(4-methylpiperazin-1-yl)methanone	21	7.1891	7.39202	-0.20292	0.255295
**29**	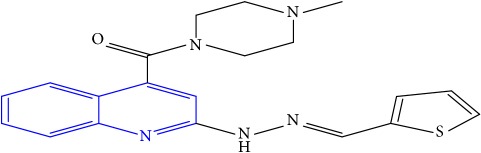 (E)-(4-methylpiperazin-1-yl)(2-(2-(thiophen-2-ylmethylene)hydrazinyl)quinolin-4-yl)methanone	10	6.8291	6.508441	0.320659	0.06229
**30**	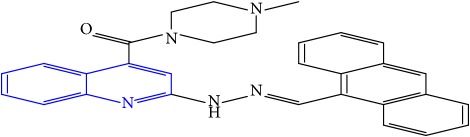 (E)-(2-(2-(anthracen-9-ylmethylene)hydrazinyl)quinolin-4-yl)(4-methylpiperazin-1-yl)methanone	10	6.9253	6.914677	0.010623	0.81434
**31** ^a^	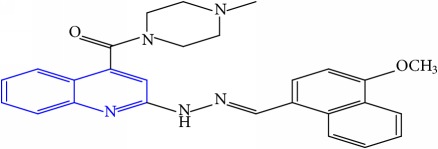 (E)-(2-(2-((4-methoxynaphthalen-1-yl)methylene)hydrazinyl)quinolin-4-yl)(4-methylpiperazin-1-yl)methanone	18	7.2022	7.50052	-0.29832	0.279776
**32**	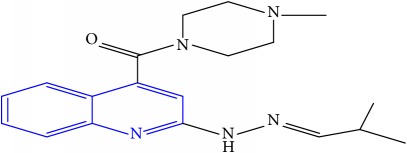 (E)-(4-methylpiperazin-1-yl)(2-(2-(2-methylpropylidene)hydrazinyl)quinolin-4-yl)methanone	52	7.7696	7.486908	0.282692	0.409976
**33**	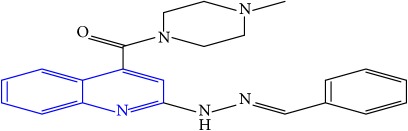 (E)-(2-(2-benzylidenehydrazinyl)quinolin-4-yl)(4-methylpiperazin-1-yl)methanone	9	6.7716	7.25273	-0.48113	0.25708
**34**	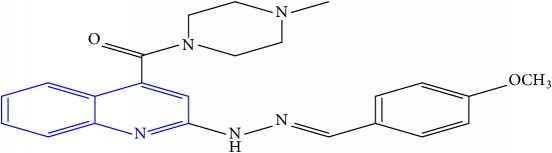 (E)-(2-(2-(4-methoxybenzylidene)hydrazinyl)quinolin-4-yl)(4-methylpiperazin-1-yl)methanone	30	7.4420	7.49224	-0.05024	0.055855
**35**	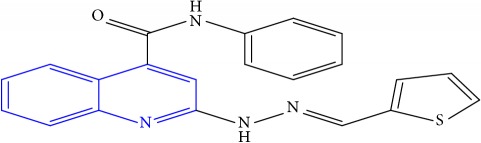 (E)-N-phenyl-2-(2-(thiophen-2-ylmethylene)hydrazinyl)quinoline-4-carboxamide	26	7.3209	7.025132	0.295768	0.517231
**36**	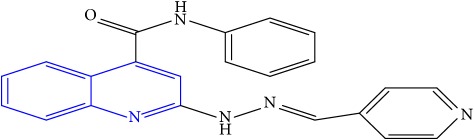 (E)-N-phenyl-2-(2-(pyridin-4-ylmethylene)hydrazinyl)quinoline-4-carboxamide	14	6.9809	7.16429	-0.18339	0.249575

Note. Superscript “a” represents the test set.

**Table 2:** Calculated descriptors for training set in generating model 1.

Molecule	Descriptor					Calculated Activity
	**AATS5e**	**VR1_Dzs**	**SpMin7_Bhe**	**TDB9e**	**RDF110s**	
*Training set*						
**10**	2.311547	0.504055	64.51552	0.52720052	0.3506263	7.67865
**11**	2.67309	0	62.68136	34.2771775	9.04275631	7.71263
**12**	2.520833	0.501468	57.73972	1.29188967	2.96E-69	6.495725
**13**	2.070513	0.399144	57.39682	2.43835699	0.19620218	9.62779
**14**	4.712551	0.452852	66.02774	6.52104829	0.35850313	7.88645
**15**	2.834823	0.442816	68.01063	4.11533689	3.17070944	7.411826
**17**	2.250086	0.432569	69.73224	4.34519754	2.69686082	7.17282
**18**	1.96649	0.413777	63.86202	0.96785765	0.09769294	7.224153
**2**	1.739712	0.413777	62.70525	4.06551831	1.08768086	6.713561
**22**	2.017931	0.413777	57.96774	3.16024723	4.02E-05	6.3256
**23**	3.22053	0.467485	63.01904	6.86345924	5.29270652	7.73765
**25**	2.44322	0.451824	59.42026	18.6036361	1.72012023	6.039603
**26**	1.951968	0.504055	63.87078	2.64230219	0.48013813	6.809542
**28**	2.25	0.41119	52.98339	1.4003672	1.32E-178	7.39202
**29**	2.136752	0.41119	56.14089	1.68288294	1.32E-85	6.508441
**3**	2.540368	0.449237	62.49834	6.73439658	1.56941859	6.664744
**30**	2.33007	0.438991	61.12375	3.13665526	0.39982877	6.914677
**32**	2.282051	0.717269	69.05135	0.80040463	2.87E-17	7.486908
**33**	4.491667	0.717269	70.41345	2.29283468	1.64E-05	7.25273
**34**	2.69287	0	59.10399	37.2430978	3.36924597	7.49224
**35**	4.934998	0.755316	72.89643	8.05935217	1.92231371	7.025132
**36**	4.808826	0.745069	77.78529	8.30282769	0.38052686	7.16429
**4**	2.177338	0.504055	60.25478	2.27249229	0.00190267	9.73193
**5**	2.497643	0	63.1492	13.5710409	4.02422392	6.896778
**6**	2.329602	0.423236	57.11063	3.94385694	0.2244206	6.510442
*Test set*						
**1**	1.843137	0.399144	58.85983	0.588352	7.75E-101	7.22456
**16**	2.535225	0	66.26276	8.996374	2.6504165	6.781862
**19**	2.16617	0.441577	58.02257	9.241266	0.6230199	7.67409
**20**	3.573278	0.464899	63.50165	5.442846	2.6206016	7.3187
**21**	6.729842	0.770977	78.82503	7.631746	6.2504921	7.273758
**24**	2.223039	0.501468	58.21113	9.046209	0.0037305	5.816571
**27**	2.031111	0.41119	56.34657	5.880833	0.2562624	7.357741
**31**	2.499622	0.422785	59.88793	2.565246	0.22884	7.50052
**7**	2.911765	0.501468	55.17425	1.262144	2.64E-182	6.972982
**8**	1.571429	0.588889	0	6.09E-17	4.24E-298	7.152527
**9**	2.568603	0	63.93143	7.576514	1.2281457	6.985668

**Table 3:** List of some descriptors used in the QSAR optimization model.

S/NO	Descriptors symbols	Name of descriptor(s)	Class
**1**	**AATS5e**	Average Broto-Moreau autocorrelation - lag 5 / weighted by Sanderson electronegativities	2D
**2**	**VR1_Dzs**	Randic-like eigenvector-based index from Barysz matrix / weighted by I-state	2D
**3**	**SpMin7_Bhe**	Smallest absolute eigenvalue of Burden modified matrix - n 7 / weighted by relative Sanderson electronegativities	2D
**4**	**TDB9e**	3D topological distance based autocorrelation - lag 9 / weighted by Sanderson electronegativities	3D
**5**	**RDF110s**	Radial distribution function - 110 / weighted by relative I-state	3D

**Table 4:** Statistical parameters that influence the model.

Descriptor	Standard regression coefficient (*b*_*j*_)	Mean Effect (ME)	P- Value (Confidence interval)	VIF	Standard Error
**AATS5e**	-0.3532	-0.4429	0.000546	2.1943	0.00654
**VR1_Dzs**	0.2376	0.3552	0.0236	2.3743	0.53182
**SpMin7_Bhe **	-0.1343	-0.8826	4.34E-04	1.6456	0.7866E-05
**TDB9e**	0.5789	0.5196	2.12E-05	1.0491	0.00867
**RDF110s**	0.94224	-0.4405	0.0135	2.7860	3.65E-05

**Table 5:** Pearson's correlation coefficient for the descriptor used in the QSAR model.

Inter-correlation					
	***AATS5e***	**VR1_Dzs**	**SpMin7_Bhe**	**TDB9e**	**RDF110s**
**AATS5e**	1				
**VR1_Dzs**	0.414812	1			
**SpMin7_Bhe**	0.668151	0.498043	1		
**TDB9e**	0.1092	-0.67462	-0.04264	1	
**RDF110s**	0.061763	-0.6067	0.095274	0.0728009	1

**Table 6:** Validation parameters for each model using multilinear regression (MLR).

S/NO	Validation Parameters	Formula	Threshold	Model 1	Model 2	Model 3	Model 4
*Internal Validation*							
**1**	Friedman LOF	$\frac{SEE}{{\left(1-\left(C+d\times p\right)/M\right)}^{2}}$		0.03167	0.03253	0.03561	0.04567
**2**	R-squared	$1-\left[\frac{\sum {\left(Y_{exp-Y_{pred}}\right)}^{2}}{\sum {\left(Y_{exp-{\bar{Y}}_{training}}\right)}^{2}}\right]$	R^2^ > 0.6	0.9265	0.8765	0.8454	0.8123
**3**	Adjusted R-squared	$\frac{R^{2}-P\left(n-1\right)}{n-p+1}$	R_adj_^2^ > 0.6	0.9045	0.8464	0.8277	0.7800
**4**	Cross validated R-squared (*Q*_*cv*_^2^)	$1-\left[\frac{\sum {\left(Y_{pred-Y_{exp}}\right)}^{2}}{\sum {\left(Y_{exp-{\bar{Y}}_{training}}\right)}^{2}}\right]$	Q^2^ > 0.6	0.8512	0.8154	0.7574	0.7245
**5**	Significant Regression			Yes	Yes	Yes	Yes
**6**	Critical SOR F-value (95%)	$\frac{\sum {\left(Y_{pred-Y_{exp}}\right)}^{2}}{p}/\frac{\sum {\left(Y_{pred-Y_{exp}}\right)}^{2}}{N-p-1}$	F_(test)_ > 2.09	3.6465	3.6542	3.75443	3.8743
**7**	Replicate points			0	0	0	0
**8**	Computed observed error			0	0	0	0
**9**	Min expt. error for non-significant LOF (95%)			0.03432	0.0354	0.04632	0.0485
*Model Randomization*							
**10**	Average of the correlation coefficient for randomized data (${\overset{-}{R}}_{r}$)		$\overset{-}{R}<0.5$	0.3866	0.3265	0.4644	0.4875
**11**	Average of determination coefficient for randomized data ( ${\overset{-}{R}}_{r}^{2})$		${\overset{-}{R}}_{r}^{2}<0.5$	0.1465	0.1843	0.2541	0.2533
**12**	Average of leave one out cross-validated determination coefficient for randomized data ( ${\overset{-}{Q}}_{r}^{2}$ )		${\overset{-}{Q}}_{r}^{2}<0.5$	-1.3325	-1.3522	-1.4023	-1.4854
**13**	Coefficient for Y-randomization (c*R*_*p*_^2^)	$R^{2}\times \;\left(1-\sqrt{\left|R^{2}-{\overset{-}{R}}_{r}^{2}\right|}\;\right)$	^c^R_p_^2^ > 0.6	0.7443	0.7103	0.6587	0.5873
*External validation*							
**14**	Slope of the plot of Observed activity against Calculated activity values at zero intercept (**K**)	$\frac{∆Y_{Obs}}{{∆Y}_{cal}}$	0.85<k<1.15	1.0016	1.04732	1.0054	1.1134
**15**	Slope of the plot of Calculated against Observed activity at zero intercept (**k**′)	$\frac{∆Y_{Cal}}{∆Y_{Obs}}$	0.85<k<1.15	0.81233	0.9432	0.6432	0.96433
**16**	/**r**_0_^2^ − **r**′_0_^2^/		<0.3	0.01643	0.07433	0.05322	0.04324
**17**	$\frac{r^{2}-r_{0}^{2}}{r^{2}}$		<0.1	0.00243	0.00573	0.07843	0.0643
**18**	$\frac{r^{2}-{r^{\prime }}_{0}^{2}}{r^{2}}$		<0.1	0.05332	0.06453	0.07637	0.8633
**19**	**R**_**test**_^2^	$1-\;\frac{\sum {\left(Y_{ext}-{\hat{Y}}_{ext}\right)}^{2}}{\sum {\left(Y_{ext}-\overset{-}{Y}\right)}^{2}}$	R_pred_^2^ > 0.6	0.8034	0.75433	0.6765	0.6123

**Table 7:** Y-randomization parameters test.

Model	**R**	**R** ^∧^2	**Q** ^∧^2
Original	0.9265	0.9045	0.8512
Random 1	0.3454	0.1193	-1.0841
Random 2	0.4868	0.2370	-1.0985
Random 3	0.4408	0.1943	-0.9815
Random 4	0.5575	0.3108	-0.5503
Random 5	0.2957	0.0874	-1.1088
Random 6	0.5562	0.3093	-0.7285
Random 7	0.7724	0.5966	0.0328
Random 8	0.2752	0.0757	-1.1166
Random 9	0.74823	0.5598	-0.0362
Random 10	0.5557	0.3088	-0.4448
*Random Models Parameters*			
Average **r**:	0.3866		
Average **r**^∧^2:	0.1465		
Average **Q**^∧^2:	-0.3325		
**c** **R** **p** ^∧^2:	0.7443		

**Table 8:** D optimal validation parameters.

D optimal Validation parameters	Value
Correlation Coefficient	0.899599
R-squared	80.9278 percent
R-squared (adjusted for d.f.)	80.0986 percent
Standard Error of Est.	0.345508
Mean absolute error	0.25514
Durbin-Watson statistic	1.81474 (P=0.3302)
Lag 1 residual autocorrelation	0.0925989
Correlation Coefficient	0.899599

## Data Availability

The derivatives of 2,4-disubstituted quinoline as potent anti-*Mycobacterium tuberculosis* that were used in this research were selected from the literature [3].
